# Lycopene Enhances Antioxidant Enzyme Activities and Immunity Function in *N*-Methyl-*N*′-nitro-*N*-nitrosoguanidine–Induced Gastric Cancer Rats

**DOI:** 10.3390/ijms12053340

**Published:** 2011-05-23

**Authors:** Cong Luo, Xian-Guo Wu

**Affiliations:** 1 Chemotherapy Department, Zhejiang Cancer Hospital, Hangzhou City, Zhejiang, 310022, China; E-Mail: lucongzjzlyy@163.com; 2 Department of Clinical Laboratory, Second Affiliated Hospital, Zhejiang University School of Medicine, 88 Jiefang Road, Hangzhou City, Zhejiang, 310009, China

**Keywords:** lycopene, MNNG, gastric cancer, digestive tract, rat

## Abstract

To investigate anticancer effect of lycopene, we examined the effects of lycopene on the oxidative injury and immunity activities of *N*-methyl-*N*′-nitro-*N*-nitrosoguanidine (MNNG)-induced gastric cancer rats. The animals were divided into five groups. Group I served as the normal control and was given corn oil orally for 20 weeks. Group II were induced with MNNG 200 mg/kg body weight by oral gavage at days 0 and 14, and saturated NaCl (1 mL per rats) was given once every three days for four weeks until the end of the experimental period. Group III, IV and V were posttreated with lycopene (50, 100 and 150 mg/kg body weight, dissolved in corn oil) from the sixth week of MNNG (as in group II) induction up to the end of the experimental period. In the presence of MNNG, MDA and immunity levels were significantly increased, whereas enzymatic (SOD, CAT, and GPx) antioxidant activities were decreased in the treated rats compared with normal control rats. Administration of lycopene to gastric carcinoma-induced rats largely up-regulated the redox status and immunity activities to decrease the risk of cancer compared to group II. We conclude that up-regulation of antioxidants and immunity by lycopene treatment might be responsible for the anticancer effect in gastric carcinoma.

## 1. Introduction

During the past five decades, the incidence and mortality rates for gastric cancer (GC) have decreased worldwide, especially in developed countries [1]. Nevertheless, GC is still the second largest cause of cancer-related death, and the overall outcome for patients with GC has not improved significantly over recent decades [2]. *N*-Methyl-*N*′-nitro-nitrosoguanidine (MNNG) is a gastric carcinogen in several animal species and has been used in a number of systems to dissect the cocarcinogenic potential of various compounds in the induction of gastric adenocarcinoma. The existence of the mechanism of action of MNNG is thought to be due to its decomposition to short-lived highly reactive electrophiles, of which the alkonium ion is probably the ultimate mutagen, and electrophilic attack on nucleophilic sites of DNA bases that leads to altered bases [3]. In addition, sodium chloride (NaCl) enhances both the initiation and promotion stages of experimental gastric carcinogenesis in rats [4–6].

Reactive oxygen species-mediated tissue injury is a final common pathway for a myriad of disease processes [7,8]. The body is continuously exposed to free radicals and ROS, both from external sources (sunlight, other forms of radiation, pollution) and endogenously generated sources. Oxidative stress can cause cancer [9], and it has been reported that the gastrointestinal tract is thought to be, at the same time, a major site for oxidant production and also a source of antioxidants. Oxidative stress can modulate the apoptotic program [10] and could lead to gastric cancer [11].

Effective immunity protects against pathogens, enables wound healing and tissue repair, and defends against some types of tumors. Tumor cells can evade immune detection and elimination by various mechanisms [12]. When this occurs, robust immunity is still essential for maintaining the general health of the cancer patient.

Phenolic compounds are widely present in plants and have recently received considerable attention due to their antioxidant and anti-inflammatory properties. Lycopene, a carotenoid mostly found in tomatoes and tomato products, is a powerful antioxidant with a singlet-oxygen-quenching capacity 47 and 100 times greater than that of β-carotene and vitamin E respectively [13,14]. Lycopene is also a potent neuroprotective [15], antiproliferative, anticancer [16–19], anti-inflammatory, cognition enhancer [20] and hypocholesterolemic agent [21–23].

In the present study, we report on the antioxidant and immunity activities of the lycopene in gastric cancer rats.

## 2. Methods

### 2.1. Extraction of Lycopene

Fifty grams of fresh and finely ground tomato peels were placed into amber flasks, to which 500 mL of 0.05% (w/v) BHT in acetone, 500 mL of ethanol and 250 mL of hexane were rapidly added. The flasks were magnetically stirred for 15 min, after which time 400 mL of deionized water were poured into the flask to allow phase separation to occur. The flasks were stirred continuously for 5 min and then the system was left at room temperature for further 5 min. Finally, a sample of the upper hexane layer was taken and analyzed for lycopene content. The process was repeated three times and the total lycopene content was calculated as the sum of the values obtained in each extraction stage. HPLC analysis showed that the purity of lycopene was 96%.

### 2.2. Animals

A total of 40, 6-week-old, male Wistar rats, were supplied by laboratory animal center of Zhejiang Chinese Medical University. The rats were raised in an air-conditioned room under controlled lighting (12 h lighting/day) and provided with food and water at discretion. Animal care and surgery protocols were approved by the Animal Care Committees of China. All animals were appropriately conducted and used in a scientifically valid and ethical manner.

### 2.3. Experimental Design

The rats received a standard diet (energy, 15.1591 kJ/kg; crude protein, 22.08%; crude fat, 4.10%; crude fiber, 3.15%; ash, 5.14%; sand silica, 1.13%) and were divided into 5 groups of 8 animals each. Group I served as the normal control and was given corn oil orally for 20 weeks. Group II were induced with MNNG 200 mg/kg body weight once daily by oral gavage for two weeks, and saturated NaCl (1 mL per rat) was then given once every 3 days for 4 weeks and then maintained until the end of the experimental period. Group III, IV and V were posttreated with lycopene (50, 100 and 150 mg/kg body weight, dissolved in corn oil) once daily from the sixth week of MNNG (as in group II) induction up to the end of the experimental period. The body weights were measured at the end of experiment. The experiment was terminated in the 21st week, and all rats were killed after an overnight fast. Blood was collected, and the plasma separated was used for analysis. The stomachs were excised to prepare a 10% homogenate for biochemical measurements. Frozen gastric tissue was ground in liquid nitrogen and suspended in a homogenization buffer consisting of 50 mM Tris–HCl, pH 8.2, 1 mM EDTA, 0.1% Triton X-100, and proteinase inhibitor cocktail (Roche, Mannheim, Germany). After centrifugation in a microcentrifuge at 4 °C, the supernatants were used to determine enzyme activity and protein concentration.

### 2.4. Biochemical Analysis

#### 2.4.1. SOD Activity Assay

The SOD activity was spectrophotometrically measured using the method developed by Marklund and Marklund [24]. Briefly, SOD was detected on the basis of its ability to inhibit superoxide-mediated reduction. One unit was determined as the amount of enzyme that inhibited oxidation of pyrogallol by 50%. The activity was expressed as U/mg protein.

#### 2.4.2. CAT Activity Assay

Catalase (CAT) activity was measured according to the Aebi [25] method. One unit of CAT activity was defined as the amount of enzyme required to decompose 1 μmol of H_2_O_2_ in 1 min. In a cuvette containing 1.95 mL of a 50 mM phosphate buffer (pH 7.0), 0.05 mL of tissue supernatant was added. The reaction was started by the addition of 1.0 mL of freshly prepared 30 mM H_2_O_2_. The rate of decomposition of H_2_O_2_ was measured spectrophotometrically at 240 nm for 1 min. Using the reaction time (Δt) of the absorbance (A1 and A2), the following equation was generated to calculate the rate constant (k): k = (2.3/Δt)(log A1/A2). The enzyme activity was expressed as k mg^−1^ protein.

#### 2.4.3. Assay of Glutathione Peroxidase Activity

Glutathione peroxidase (GPx) activity was measured by the procedure of Flohe and Gunzler [26]. One milliliter of reaction mixture that contained 0.3 mL of phosphate buffer (0.1 M, pH 7.4), 0.2 mL of glutathione (GSH) (2 mM), 0.1 mL of sodium azide (10 mM), 0.1 mL of H_2_O_2_ (1 mM), and 0.3 mL of stomach supernatant was prepared. After incubation at 37 °C for 15 min, the reaction was terminated by addition of 0.5 mL 5% TCA. Tubes were centrifuged at 1500 g for 5 min and the supernatant was collected. 0.2 mL of phosphate buffer (0.1 M, pH 7.4), and 0.7 mL of 5,5′-dithio-bis- (2-nitrobenzoic acid)(DTNB, 0.4 mg/mL) were added to 0.1 mL of reaction supernatant. After mixing, absorbance was recorded at 420 nm and the enzyme activity was calculated as U/mg protein.

#### 2.4.4. The Tissues MDA Concentration

The tissues MDA concentration was determined using the method described by Jain *et al.* [27], based on TBA reactivity. Briefly, 0.2 mL supernatant obtained from tissues, 0.8 mL phosphate buffer (pH 7.4), 0.025 mL BHT and 0.5 mL 30% TCA were added to the tubes and mixed. After 2 h incubation at −20 °C, the mixture was centrifuged (4000 × g) for 15 min. After this, 1 mL supernatant was taken and added to each tube, and then 0.075 mL of 0.1 M EDTA and 0.25 mL of 1% TBA were added. These tubes with Teflon-lined screw caps were incubated at 90 °C in a water bath for 15 min and cooled to room temperature. The optical density was measured at 532 for tissues MDA concentration.

#### 2.4.5. IL-2, IL-4, IL-6, IL-10, TNF-α, IgA, IgG and IgM Levels

The concentrations of interleukin-2 (IL-2), interleukin-6 (IL-6), interleukin-4 (IL-4), and interleukin-10 (IL-10) in the supernatant were determined using appropriate commercial ELISA kits for the murine form of these cytokines. ELISAs were performed according to the manufacturers’ recommendations. The intensity of each sample was read at 450nm in a microplate spectrophotometer.

Plasma tumor necrosis factor alpha (TNF-α) level was determined by commercial ELISA kits according to the protocol recommended by the manufactures. The results were expressed as the quantity per ml plasma.

Blood IgA, IgM and IgG levels were measured with a commercially available ELISA kits.

### 2.5. Statistical Analysis

All data are presented as means ± SD. Statistical analyses were done using SPSS (Statistical Package for Social Science, SPSS Inc., Chicago, IL, USA) program, and significance of each group was verified with one-way ANOVA followed by Duncan’s test (p < 0.05).

## 3. Results

In the normal group, the rats looked perfectly relaxed, moved quickly and ate well. Their hair looked bright, and faeces was hard and granular. In the MNNG-treated groups, the rats looked less lively or vigorous, moved slowly, ate less and had diarrhea. Their hair looked loose and lusterless. The body weights of rats in the model group were significantly lower than those in the normal control group. Pathological examination showed that MNNG-treated rats had varying degrees of atrophy and dysplasia on histological examination of the gastric mucosa. These indicated that gastric cancer model had been successfully established (data not shown).

There was no marked difference in body weight between groups before experiment. After experiment, the average body weight of the gastric cancer model rats was significantly (p < 0.05) reduced compared to that of the normal control rats. Lycopene treatment significant (p < 0.05) increased the body weight of the gastric cancer rats (Table 1).

Compared with the normal control rats, significantly (p < 0.01) increased MDA levels of blood and gastric tissues were observed in the gastric cancer model rats (Tables 2 and 3). The gastric cancer rats exhibited an increased MDA level at the end of the experiment; this state was significantly ameliorated by the lycopene treatment. The activities of SOD, CAT and GSH-Px in the blood and gastric tissue were significantly (p < 0.01) decreased by MNNG treatment in comparison with the normal control rats. Similarly, the administration of the lycopene significantly (p < 0.01) enhanced the activities of antioxidant enzymes in blood and gastric tissues of MNNG-treatment rats.

One-way ANOVA showed an overall significant effect of drug treatment on blood IL-2, IL-4, IL-6, IL-10 and TNF-α levels (Table 4). Compared with the normal control rats, blood IL-2, IL-4, IL-10 and TNF-α levels were significantly reduced in the gastric cancer model rats, while blood IL-6 level was significantly increased. One-way ANOVA revealed that lycopene treatment significantly enhanced blood IL-2, IL-4, IL-10, TNF-α levels and reduced IL-6 level in a dose-dependent manner.

Compared with the normal control rats, significantly decreased blood IgG, IgA and IgM levels were observed in the gastric cancer model rats (Table 5). Pharmacological test showed that the treatment of animals with lycopene elevated blood IgG, IgA and IgM levels compared to gastric cancer model rats in a dose-dependent manner.

## 4. Discussion

Lycopene has been implicated as having a potentially beneficial impact in a number of chronic diseases including cancer. Evidence from epidemiological and animal studies supports a potential chemopreventive role of lycopene [28–30]. Studies have shown the anti-cancer properties of lycopene against many cancer cells, including the cancer cells of the stomach [31], lung [32], and colon [19].

In this study, a significant decrease in average body weight was observed in the cancer-induced rats. The subsequent increase in body weight upon administration of lycopene could be because of the protective efficacy of the lycopene. Because weight loss in cancer patients results in a poor prognosis, body weight restoration upon lycopene administration suggests a potential therapeutic benefit. Free radicals do not really affect weight loss, but they do harm your overall health. If you’re not healthy, then weight loss is kind of a moot point. The substantial weight loss observed in cancer-bearing rats could be because of cancer cachexia, anorexia, or malabsorption [33], which reportedly contributes to progressive wasting, notably in skeletal muscle and adipose tissue. Lycopene may possess an excellent antitumor potential and antioxidant benefit, which might improve rats’ health and digestive function. Subsequently, the increase in food intake might have facilitated the rise in body weight.

The detrimental effect of reactive oxygen species (ROS) has been examined in gastric tissue in detail; in addition to prostaglandin (PG) reduction, the increase in ROS levels is also important to the basic mechanism of gastric damage formation [34]. This state of oxidative stress can result in injury to all the important cellular components like proteins, DNA and membrane lipids which can cause cell death. Oxidants are capable of stimulating cell division, which is a critical factor in mutagenesis. When a cell with a damaged DNA strand divides, cell metabolism and duplication becomes deranged. Thus, a mutation can arise, which is an important factor in carcinogenesis. In recent years, increasing experimental and clinical data has provided compelling evidence for the involvement of oxidative stress in large number of pathological states including carcinogenesis [35,36]. It is believed that antioxidants exert their protective effect by decreasing oxidative damage to DNA and by decreasing abnormal increases in cell division. Therefore, it is believed that the protective functions of antioxidant mechanisms play a role in the treatment of ulcers of the gastric mucosa [37]. Healthy tissue cells have many mechanisms that can prevent the detrimental effects of ROS or can repair existing damage—primarily enzymes, and secondarily, glutathione, melatonin and antioxidant vitamins, prevent tissue damage by maintaining ROS at physiologic concentration in cells [38].

In this study, we evaluated the oxidant and antioxidant parameters of blood and gastric tissues in gastric cancer rats. Significantly increased MDA level and decreased antioxidant enzyme activities were detected in gastric cancer rats. This means that there may be a relation between gastric cancer and gastric oxidant and antioxidant parameters. The administration of lycopene at 100, 200 and 300 mg/kg doses decreased the level of oxidant parameters (MDA) and increased blood and gastric antioxidant parameters (SOD, CAT and GSH-Px) significantly. CAT, SOD and other antioxidants are endogenous factors that reduce toxic effects of ROS [39,40]. So, we can hypothesize that lycopene may reduce oxidative injury of gastric cancer rats partly through stimulating antioxidant enzyme activities. Subsequently, it may alleviate gastric cancer symptoms.

Many gastric cancer patients have a variable degree of immunological impairment, including decreased cellular immunity [41,42]. Cytokines such as interferons, interleukin and tumor necrosis factor have been widely tested in the treatment of cancer. Interleukin-2 (IL-2) or aldesleukin is used to treat skin melanomas and kidney cancer. IL-4 has been shown to have a modest but direct inhibitory effect on the growth of tumor cells of hematopoietic and nonhematopoietic origin *in vitro* and *in vivo*, including those derived from human melanoma, non-Hodgkin’s malignant B-lymphoma, and colon, renal, gastric, and breast carcinoma [43–47]. IL-6 is an interleukin that acts as both a pro-inflammatory and anti-inflammatory cytokine. It is secreted by T cells and macrophages to stimulate immune response to trauma, especially burns or other tissue damage leading to inflammation. Advanced/metastatic cancer patients have higher levels of IL-6 in their blood [48]. Hence, there is an interest in developing anti-IL-6 agents as therapy against many of these diseases [49,50]. Interleukin-10 (IL-10) is a small protein known as a cytokine that functions as an important regulator of the immune system. Although IL-10 is known to have many different roles in the immune system, its two major activities include inhibition of cytokine production by macrophages and inhibition of the accessory functions of macrophages during T cell activation [51]. The effects of these actions cause IL-10 to play an anti-inflammatory role in the immune system. TNF-α contributes to several pathophysiological states initiated by infectious or inflammatory agents by producing ROS and inducing pro-inflammatory chemoattractant cytokine cascades. A regulatory role of TNF-α in epithelial cell repair has been described [52,53]. It is well established that TNF-α stimulates IL-8 and contributes to epithelial cell injury and apoptosis [54–56]. In this study, blood IL-2, IL-4, IL-10 and TNF-α levels in gastric cancer model control rats were significantly lower than those in normal control rats. Blood IL-6 level in gastric cancer model control rats was markedly higher than that in normal control rats. This indicated that immunity function had decreased in gastric cancer rats. Lycopene treatment had significantly increased blood IL-2, IL-4, IL-10, TNF-α levels and reduced the IL-6 level in gastric cancer rats. In addition, Lycopene treatment could still enhance blood IgA, IgG and IgM levels in gastric cancer rats. These results suggest that Lycopene treatment could improve immunity function in gastric cancer rats.

## 5. Conclusion

The overall results of this study indicate that lycopene represents a potential source of plant drugs. At this stage of our study, we can deduce that lycopene appears to be worthy of consideration as an antioxidant, immune and antitumor medicine.

## Figures and Tables

**Table 1.** Effect of Lycopene treatment on body weight of gastric cancer rats.

**Group**	**Body weight (g)**	
	**Before experiment**	**After experiment**
I	187.5 ± 15.3	223.1 ± 19.4
II	188.2 ± 17.8	202.3 ± 22.1^a^
III	185.2 ± 16.2	209.4 ± 18.6
IV	184.8 ± 19.1	211.5 ± 23.5^c^
V	186.7 ± 19.3	218.2 ± 20.6^c^

Values are given as Mean ± SD for groups of eight rats each.

a p < 0.05, gastric cancer model rats (group II) were compared with normal control (group I);

c p < 0.05, lycopene treated rats (group III, IV and V) were compared with gastric cancer model control (group II).

**Table 2.** Effect of Lycopene treatment on blood SOD, CAT, GSH-Px activities and MDA level in gastric cancer rats.

**Group**	**SOD (U/mg)**	**CAT (U/mg)**	**GSH-Px (U/mg)**	**MDA (U/mg)**
I	182.16 ± 17.29	29.85 ± 2.17	31.63 ± 3.72	8.27 ± 0.69
II	98.26 ± 7.27^b^	16.72 ± 1.99^b^	18.92 ± 2.41^b^	12.18 ± 0.93^b^
III	126.13 ± 13.83^d^	19.82 ± 1.68^d^	23.86 ± 2.83^d^	10.73 ± 0.82^c^
IV	162.95 ± 19.04^d^	22.47 ± 3.01^d^	27.91 ± 3.09^d^	9.32 ± 0.79^d^
V	188.29 ± 20.11^d^	28.14 ± 3.12^d^	30.61 ± 3.21^d^	8.05 ± 0.91^d^

Values are given as Mean ± SD for groups of eight rats each.

b p < 0.05, gastric cancer model rats (group II) were compared with normal control (group I);

c p < 0.05,

d p < 0.01, lycopene treated rats (group III, IV and V) were compared with gastric cancer model control (group II).

**Table 3.** Effect of Lycopene treatment on gastric SOD, CAT, GSH-Px activities and MDA level in gastric cancer rats.

**Group**	**SOD (U/mg)**	**CAT (U/mg)**	**GSH-Px (U/mg)**	**MDA (U/mg)**
I	200.14 ± 27.31	38.12 ± 4.42	27.29 ± 3.28	6.39 ± 0.72
II	117.32 ± 14.21^b^	21.72 ± 3.17^b^	20.13 ± 2.41^b^	9.31 ± 1.32^b^
III	152.71 ± 17.08^d^	26.92 ± 3.05^d^	23.81 ± 2.64^c^	8.14 ± 0.93^c^
IV	170.48 ± 19.36^d^	29.33 ± 3.14^d^	26.18 ± 2.91^d^	7.53 ± 0.84^d^
V	194.28 ± 22.13^d^	37.11 ± 4.25^d^	29.52 ± 3.28^d^	6.54 ± 0.74^d^

Values are given as Mean ± SD for groups of eight rats each.

b p < 0.05, gastric cancer model rats (group II) were compared with normal control (group I);

c p < 0.05,

d p < 0.01, lycopene treated rats (group III, IV and V) were compared with gastric cancer model control (group II).

**Table 4.** Effect of Lycopene treatment on blood IL-2, IL-4, IL-10, IL-6 and TNF-α levels in gastric cancer rats.

**Group**	**IL-2 (ng/mL)**	**IL-4 (ng/mL)**	**IL-10 (ng/mL)**	**IL-6 (ng/mL)**	**TNF-α (ng/mL)**
I	6.04 ± 0.83	21.54 ± 1.83	90.91 ± 10.63	70.63 ± 8.42	4.13 ± 0.67
II	4.83 ± 0.51^b^	15.62 ± 1.72^b^	62.76 ± 3.84^b^	89.17 ± 9.59^b^	2.69 ± 0.49^b^
III	5.42 ± 0.63^c^	17.42 ± 2.08^c^	74.93 ± 6.20^d^	82.61 ± 10.07^c^	3.08 ± 0.44^d^
IV	5.82 ± 0.62^d^	18.96 ± 2.11^d^	82.17 ± 5.81^d^	78.49 ± 9.48^d^	3.51 ± 0.51^d^
V	6.11 ± 0.73^d^	20.51 ± 2.54^d^	89.53 ± 5.39^d^	75.37 ± 8.37^d^	3.99 ± 0.53^d^

Values are given as Mean ± SD for groups of eight rats each.

b p < 0.05, gastric cancer model rats (group II) were compared with normal control (group I);

c p < 0.05,

d p < 0.01, lycopene treated rats (group III, IV and V) were compared with gastric cancer model control (group II).

**Table 5.** Effect of Lycopene treatment on blood IgG, IgA and IgM levels in gastric cancer rats.

**Group**	**IgG (g/L)**	**IgA (g/L)**	**IgM (g/L)**
I	5.03 ± 0.31	0.64 ± 0.05	0.71 ± 0.08
II	3.52 ± 0.03^b^	0.43 ± 0.04^b^	0.55 ± 0.06^b^
III	3.97 ± 0.04^c^	0.52 ± 0.06^c^	0.62 ± 0.07^c^
IV	4.58 ± 0.05^d^	0.59 ± 0.06^d^	0.68 ± 0.05^d^
V	4.89 ± 0.06^d^	0.63 ± 0.07^d^	0.74 ± 0.06^d^

Values are given as Mean ± SD for groups of eight rats each.

b p < 0.05, gastric cancer model rats (group II) were compared with normal control (group I);

c p < 0.05,

d p < 0.01, lycopene treated rats (group III, IV and V) were compared with gastric cancer model control (group II).
